# A biophysical study on the mechanism of interactions of DOX or PTX with α-lactalbumin as a delivery carrier

**DOI:** 10.1038/s41598-018-35559-1

**Published:** 2018-11-26

**Authors:** Behdad Delavari, Fatemeh Mamashli, Bahareh Bigdeli, Atefeh Poursoleiman, Leila Karami, Zahra Zolmajd-Haghighi, Atiyeh Ghasemi, Samaneh Samaei-Daryan, Morteza Hosseini, Thomas Haertlé, Vladimir I. Muronetz, Øyvind Halskau, Ali Akbar Moosavi-Movahedi, Bahram Goliaei, Ali Hossein Rezayan, Ali Akbar Saboury

**Affiliations:** 1 Institute of Biochemistry and Biophysics, University of Tehran, Mailbox, 13145-1384 Tehran, Iran; 2 Department of Cell and Molecular Biology, Faculty of Biological Sciences, Kharazmi University, Tehran, Iran; 3 Department of Life Science Engineering, Faculty of New Sciences & Technologies, University of Tehran, Tehran, Iran; 4 Poznań University of Life Sciences, Department of Animal Nutrition and Feed Management, Poznań, Poland; 5 UR 1268 Biopolymères Interactions Assemblages, INRA, B.P. 71627, 44316 Nantes, Cedex 3 France; 6 Belozersky Institute of Physico-Chemical Biology, Lomonosov Moscow State University, 119234 Moscow, Russia; 7 Department of Molecular Biology, University of Bergen, PB 7803, N-5020 Bergen, Norway

## Abstract

Doxorubicin and paclitaxel, two hydrophobic chemotherapeutic agents, are used in cancer therapies. Presence of hydrophobic patches and a flexible fold could probably make α-Lactalbumin a suitable carrier for hydrophobic drugs. In the present study, a variety of thermodynamic, spectroscopic, computational, and cellular techniques were applied to assess α-lactalbumin potential as a carrier for doxorubicin and paclitaxel. According to isothermal titration calorimetry data, the interaction between α-lactalbumin and doxorubicin or paclitaxel is spontaneous and the K (M^−1^) value for the interaction of α-lactalbumin and paclitaxel is higher than that for doxorubicin. Differential scanning calorimetry and anisotropy results indicated formation of α-lactalbumin complexes with doxorubicin or paclitaxel. Furthermore, molecular docking and dynamic studies revealed that TRPs are not involved in α-Lac’s interaction with Doxorubicin while TRP 60 interacts with paclitaxel. Based on Pace analysis to determine protein thermal stability, doxorubicin and paclitaxel induced higher and lower thermal stability in α-lactalbumin, respectively. Besides, fluorescence lifetime measurements reflected that the interaction between α-lactalbumin with doxorubicin or paclitaxel was of static nature. Therefore, the authors hypothesized that α-lactalbumin could serve as a carrier for doxorubicin and paclitaxel by reducing cytotoxicity and apoptosis which was demonstrated during our *in vitro* cell studies.

## Introduction

In spite of intense research, cancer which claimed the lives of 8.2 million people in 2012, is still a major global threat^1,2^. Doxorubicin (DOX) and paclitaxel (PTX) are among the most effective chemotherapeutics used in the treatment of various types of cancer. DOX, an antibiotic from the anthracycline family, exhibits a broad spectrum of antineoplastic activity. In fact, DOX is one of the most powerful chemotherapeutic agents in breast cancer treatment and is used for treatment of other malignancies as well. The therapeutic activity of DOX is mainly due to its intercalation and alkylation of DNA and induction of topoisomerase II mediated strand breaks^3–5^. Another potent anticancer agent, PTX, is used for the treatment of a wide spectrum of cancers, especially ovarian and breast cancers. PTX is extracted from the bark of *Taxus brevifolia* and is a diterpenoid taxane derivative. Its unique mechanism of action is *via* binding tubulin dimers and stabilizing microtubule formation, which ultimately disrupts mitosis and results in cell death^6–8^. However, DOX and PTX are very hydrophobic and thus poorly soluble in aqueous media, which presents critical challenges for clinical application^7,9^. Therefore, several approaches have been investigated to develop novel aqueous formulations including, the use of liposomes, micelles, nanoparticles, nanocapsules, polymeric microspheres, and bioconjugation with different molecules, e.g., albumin, etc^8^. As an indication, Doxil®, the first FDA-approved nano-therapeutic agent, is a liposomal preparation of DOX effective in the treatment of ovarian and some other forms of cancers^10^. In the case of PTX, Abraxane®, an albumin bound nanoparticle, is a nanovector with special application in the treatment of breast cancer and non-small cell lung cancer^11,12^.

Various proteins have potential as nanocarriers in drug delivery, and are being considered as GRAS (generally regarded as safe)^13^. Moreover, they have a broad range of characteristics such as biodegradability, exceptional binding capacity for different drugs, as well as less opsonization by the reticuloendothelial system (RES)^14^. For instance, human serum albumin, bovine serum albumin, and the milk protein β-lactoglobulin have been studied as carriers for DOX^15,16^. In particular, milk proteins have numerous structural and functional properties which could facilitate their application as delivery agents of a variety of bioactive compounds. They can bind to ions and/or hydrophobic molecules with various degrees of affinities, and are widely available, natural, inexpensive, biocompatible, and biodegradable^17^.

α-Lactalbumin (α-Lac), the second most abundant among milk whey proteins, is an acidic (pI 4.8), low molecular weight (14.2 kD) globular protein^18,19^. α-Lac is involved in the biosynthesis of lactose as a component of lactose synthetase^20^. Hydrophilic α-Lac is present in the milk of nearly all mammals and composes 3.4% of total bovine milk proteins (1–1.5 g/l)^21^. This calcium binding metalloprotein contains two domains; a large helical domain (residues 1–34 and 86–123) and a small β sheet domain (residues 35–85) connected by a loop. The α helical domain made up of three main α helices (residues 5–11, 23–24, and 86–98) and two smaller 3_10_ helices (residues 18–20 and 115–118). The β sheet domain consists of three antiparallel β strands (residues 41–44, 47–50, and 55-56), a 3_10_ helix (residues 77-80), and several loops. There is a deep cleft between the two domains and a calcium ion is connected to its binding site in the loop connecting the two domains (in the native α-Lac). Ca^2+^-bound and -depleted forms of α-Lac are referred to as holo-α-Lac and apo-α-Lac, respectively. It is important to note that, apo-α-Lac presents altered tertiary structure compared to holo-α-Lac^22^. α-Lac also contains eight cysteine (Cys) residues, which form four disulfide bridges (6–120, 61–77, 73–91, and 28–111) and one hydrophobic pocket^21,23^. Interestingly, it has been shown that oleic and palmitic acids can only bind to apo-α-Lac which harbors one binding site for oleic acid and another for palmitic acid^24^. The complex formed by apo form of human or bovine α-Lac and oleic acid demonstrate cytotoxicity in various tumor cell lines^25^. These complexes exist in a core-shell configuration where fatty acids or other hydrophobic substances are encapsulated by several partially denatured, flexible protein chains^26,27^. α-Lac has also been shown to complex with other hydrophobic compounds, including fatty acids^28,29^, retinol^17^, hydrophobic column chromatography phases, hydrophobic peptides, melittin of bee venom, and vitamin D_3_^30–32^.

α-Lac has been applied in preparation of stable nanosystems harboring DOX via self-assembly^33^. According to the results, α-Lac and the prepared nanosystems presented less toxicity in normal compared to tumor cells. This already has been determined in the case of human α-Lac-oleic acid complex (HAMLET) and bovine α-Lac-oleic acid complex (BAMLET). α-Lac’s specificity in exerting less damages to normal cells compared to tumor cells has distinguished it among other proteins^25^. Furthermore, α-Lac, as the second whey protein, improved solubility of α-Lac than caseins, presence of binding sites for hydrophobic ligands, biocompatibility, and biodegradability make α-Lac a suitable candidate as a drug delivery agent. α-Lac is considered less immunogen compared to other major milk proteins due to eliciting lower levels of IgE. Besides, there is no dominant epitope in α-Lac in the case of T cells’ responses^34^. In the present study, we aimed to evaluate α-Lac’s potential as a carrier for chemotherapeutic agents such as DOX or PTX using thermodynamic, spectroscopic, and computational techniques. Furthermore, we are going to answer the question of whether α-Lac is capable of embracing the chemotherapeutic agents and consequently causing the slow release of the drugs and their less toxicity by performing and comparing the cellular test results. The current paper reinforces the importance of performing biophysical and thermodynamic studies before preparation of therapeutic nanocarrier.

## Results and Discussion

### Isothermal titration calorimetry

**Figure 1:**
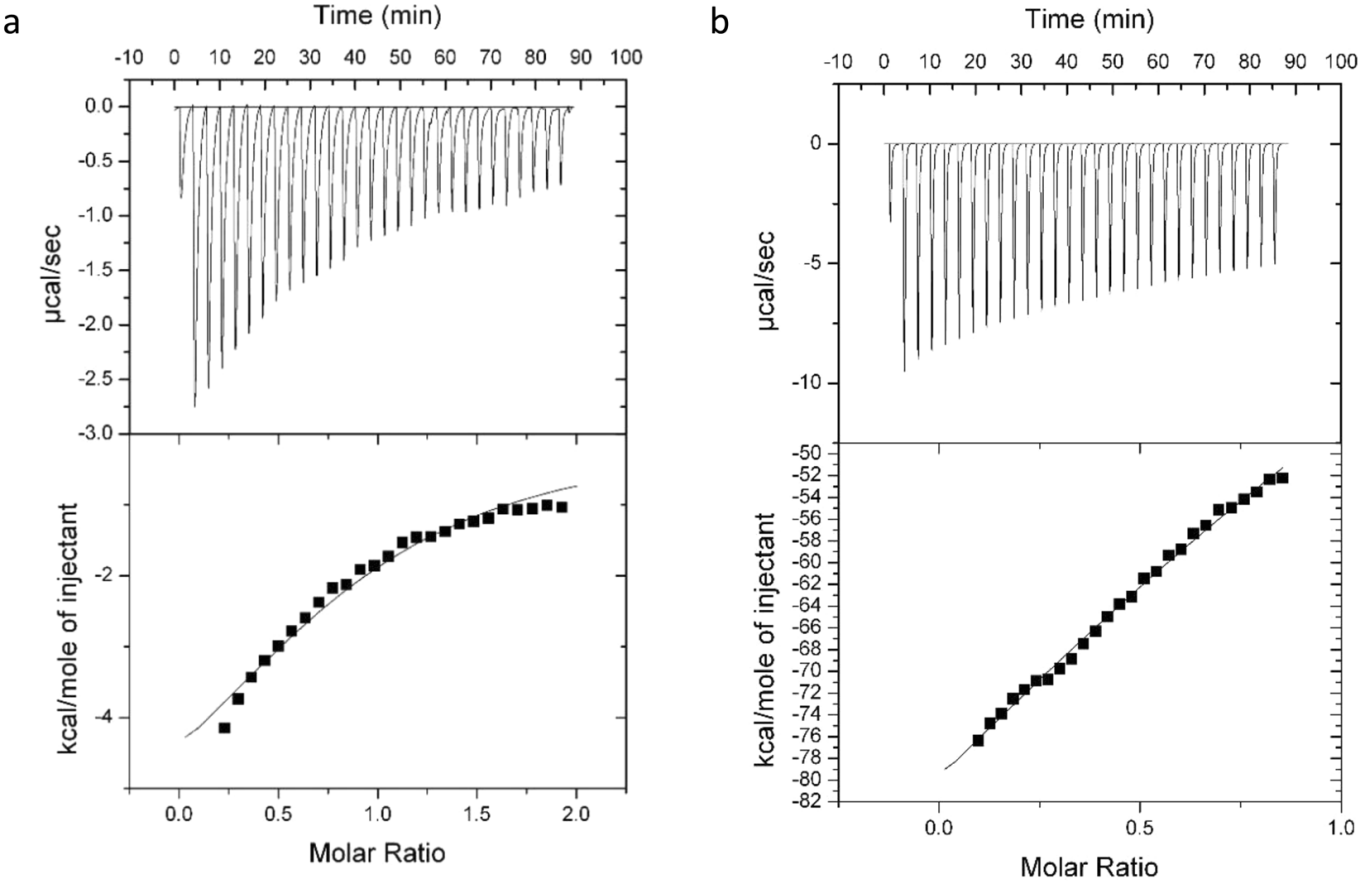
ITC diagram from the titration of α-La with (**a**) DOX and (**b**) PTX. 290 µM α-La was titrated with 3.4 mM DOX and 50 µM α-La was titrated with 250 µM PTX at 25 °C. The amount of heat measured per injection and the amount of heat measured per mole of the injected DOX or PTX as a function of the molar ratio of α-Lac to DOX or PTX for each injection are shown at top and bottom, respectively. The heat effects due to mixing and dilution were corrected by subtracting the heat effects of PTX and DOX in protein buffer and the effect of ethanol as PTX’s solvent under identical condition (temperature and timing) from the heat of α-Lac:DOX or α-Lac:PTX titration.

ITC, a powerful technique to study biomolecular interactions, provides a direct method to determine thermodynamic parameters of binding such as binding stoichiometry (*n*), standard enthalpy change (*ΔH°*), entropy change (*ΔS°*), and binding constant (*K*_*b*_) in one experiment^35^. The primary titration profile of the binding of DOX or PTX to α-Lac at 25 °C and the theoretical fits are shown in Fig. 1a,b. Furthermore, the estimated thermodynamic parameters are comprised in Table 1. Since ethanol was used to dissolve PTX, it should be noted that the α-Lac thermal changes due to 3% (v/v) ethanol (see Experimental and Theoretical Methods section) was negligible and not included in the manuscript. Although care should be taken in interpreting ITC data where there is no clear pre-transition or post-transition, it seemed clear that interaction does occur, albeit at low affinity. However, this seems natural for large molecular ligands such as DOX or PTX. Employing higher concentrations of 500 μM in the case of HAS and β-lactoglobulin which are larger proteins with more capacity to interact with DOX or PTX compared to α-Lac, K_b_ values of 1.0×10^4^–1.43×10^4^ have been reported^15,36,37^. These values are similar or a little higher than our obtained K_b_ values. In general, it can be concluded that larger ligands present lower values of K_b_ compared to smaller ligands (K_b_ values of 10^6^−10^8^).

**Table 1:** ITC Analysis of DOX or PTX’s Binding to α-Lac in 20 mM Tris Buffer (pH 7) at 25 °C.

Sample	*n*	*K* (M^-1^)	*ΔH°* (kcal/mol)	*ΔS°* (cal/mol K)	*ΔG°* (kcal/mol)
α-Lac:DOX	0.9 ± 0.104	(6.7 ± 1.61) × 10^3^	−6.8 ± 1.23	−5.37	−5.22
α-Lac:PTX	1.8 ± 0.066	(14 ± 4.82) × 10^3^	−145 ± 21	−467	−5.53

The heat released upon each injection of DOX or PTX solution is reduced gradually with each sequential injection, but does not level off completely within the practical limitations of the experiments. This may indicate not only binding of DOX or PTX, but also a reorganization of the protein complex. Near- and Far-UV CD experiments along with computational simulations indicate that this is the case (*vide infra*). Moreover, the number of initial DOX binding sites is clearly smaller than the number of initial PTX binding sites on α-Lac (Table 1). This could be due to dissimilar changes in secondary and tertiary structures in α-Lac during interactions with DOX or PTX and formation of another binding site during PTX interaction process. The value of K_b_ for α-Lac:DOX is smaller than that of α-Lac:PTX complexes, which could be resulted from larger hydrophobic forces in interaction of PTX with α-Lac than DOX. This is discussed in the results obtained by molecular dynamic studies.

According to values of *ΔH°*, both interactions are exothermic but the amount of change is enormous in the case of PTX. This could be due to a higher enthalpy in PTX interaction with α-Lac, extensive conformational changes of the protein upon interaction with PTX, strong hydrophobic protein-protein interactions and consequent formation of protein assemblies (also indicated by anisotropy and DSC, *vide infra*), two initial binding sites for PTX, and formation of PTX assemblies in water. It is of special importance to note that enthalpy of PTX solution in the protein buffer was too high, so it was minimized during numerous tests. To this end, PTX was diluted in the protein buffer and also the amount of ethanol needed to dissolve the PTX was minimized.

Based on *ΔS°* values, α-Lac:PTX presented greater reduction in entropy compared to α-Lac:DOX complexes. This could be due to protein-protein hydrophobic interactions in the presence of the ligand and alteration in arrangement of water molecules in the presence of PTX assemblies in water. Moreover, α-Lac:PTX and α-Lac:DOX interactions are both spontaneous as implied by *ΔG°* values. Furthermore, considering the negative values of *ΔH°* and *ΔS°* for α-Lac:DOX and α-Lac:PTX complexes, it could be concluded that van der Waals forces, hydrogen bonds, and protonation were the effective driving forces of interactions^38,39^.

### Differential scanning calorimetry

**Figure 2:**
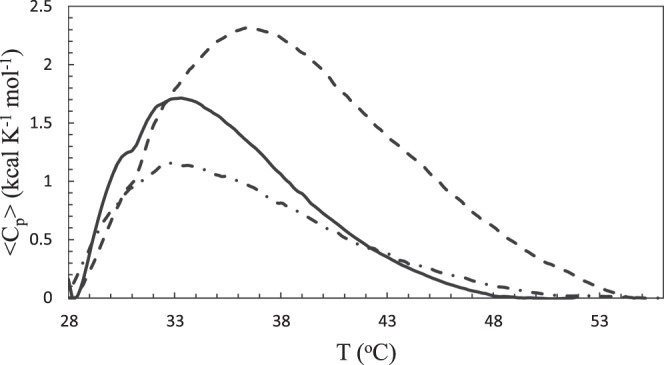
DSC thermograms of apo α-Lac in the presence of DOX and PTX. DSC thermograms of 3 mg/ml apo α-Lac in 20 mM Tris buffer (pH 7) containing 3.5 mM EDTA were obtained in the presence of 0 mM DOX (solid line), 0.86 mM DOX (dashed line), 108 µM PTX (dot dash line). The measurements were performed over a temperature range of 15–60 °C.

Heat denaturation was studied to determine the effect of DOX or PTX on the stability of α-Lac using DSC. Excess heat capacity as a function of temperature is shown in DSC thermograms (Fig. 2). Furthermore, melting temperature (*T*_*m*_), the standard enthalpy change of denaturation (*ΔH°*), the standard entropy change of denaturation (*ΔS°*), and the standard heat capacity change upon α-Lac unfolding (*ΔC°*_*p*_) were calculated according to DSC data and presented in Table 2. Presence of DOX caused increased *T*_*m*_, *ΔH°*, *ΔS°*, and *ΔC°*_*p*_ values for 1:5.5 ratio of α-Lac:DOX complex compared to α-Lac. A higher *ΔC°*_*p*_ value indicates that hydrophobic patches of α-Lac in complex with DOX are more exposed than α-Lac^40^. Moreover, *ΔH°* is thought to derive from intermolecular interactions between hydrophobic amino acids of the proteins. Thus, a higher *ΔH°* value resulted from DOX interaction with α-Lac reflects greater exposure of hydrophobic patches of α-Lac, which could cause hydrophobic interactions between α-Lac molecules and induce formation of α-Lac assemblies. Furthermore, a higher and positive value of *ΔH°* following interaction of DOX and α-Lac implies that α-Lac denaturation is an endothermic process.

**Table 2:** Thermodynamic Parameters of α-Lac’s Denaturation with DOX or PTX Determined by DSC.

Sample	*T*_*m*_ (°C)	*ΔH°* (kcal/mol)	*ΔS°* (cal/mol K)	*ΔC°*_*p*_ (kcal/mol K)
α-Lac	33.3	16.6	54	0.70
α-Lac:DOX	36.4	30.8	100	1.15
α-Lac:PTX	32.7	14.7	47	0.31

In addition, the presence of PTX resulted in a remarkably higher T_m_ value at 1:5.5 ratio of α-Lac:PTX complex than α-Lac. However, the DSC thermogram of α-Lac and PTX interaction (Fig. 1, Supplementary information) shows several peaks, which implies the presence of several populations of protein assemblies and not feasible to analyze. Therefore, we decreased the ratio of α-Lac:PTX to 1:0.5 and could obtain a DSC thermogram with one peak. Moreover, for higher concentrations of PTX, the authors employed Pace analysis.

This observation could be due to the fact that PTX is more hydrophobic and causes more hydrophobic-hydrophobic interactions among proteins in higher concentrations of PTX. According to the data presented in Table 2, no significant change is observed in the values of *T*_*m*_, *ΔH°*, and *ΔS°* parameters between α-Lac and 1:0.5 ratio of α-Lac:PTX complex. On the other hand, *ΔC°*_*P*_ data implied that a decreased value of *ΔC°*_*P*_ in lower concentrations of PTX reflects less exposure of α-Lac hydrophobic patches. This, in turn, results in reduced hydrophobic-hydrophobic interactions among proteins at lower concentrations of PTX, which is coherent with the anisotropy data.

### Circular dichroism spectroscopy

**Figure 3:**
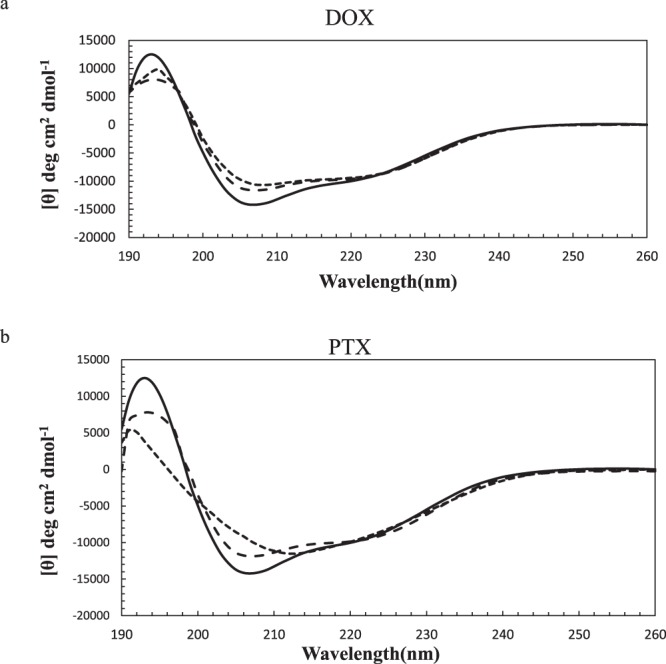
Far-UV CD spectra of α-Lac in the presence of (**a**) DOX and (**b**) PTX. 10 µM α-Lac dissolved in 20 mM Tris buffer (pH 7) was scanned in the absence (solid line) and the presence of 2 (long dashed line) and 5.5 (dashed line) fold excess concentrations of DOX or PTX. All measurements were performed at the room temperature (25 °C).

Far-UV CD was performed to monitor the secondary structural changes of α-Lac upon interaction with DOX or PTX. The diagram presented in Fig. 3a,b shows [θ] (deg cm^2^ dmol^−1^) versus wavelength (nm). As presented in the diagram, with added DOX or PTX the CD spectral profile of α-Lac underwent significant changes. According to the CD data (presented in Table 1, Supplementary information), DOX induced a reduction of α-helix content from 34.2% in the native α-Lac to 30.2% in the complex when the concentration of DOX was 2 times greater than α-Lac. Surprisingly, PTX did not induce a significant change in the α-helix content of α-Lac at this concentration. However, applying 5.5 fold excess of DOX or PTX than α-Lac reduced the α-helix and increased the random coil content which still are considered as slight changes. This effect was higher in the case of DOX and was supported by our MD results (*vide infra*).

Furthermore, secondary structural changes induced by DOX or PTX were concentration dependent and increased when measured at higher concentrations. This could indicate the perturbation of the secondary structure of α-Lac by DOX or PTX. There were no perceivable changes in the contents of parallel or anti-parallel β-sheets and β-turns. The extent of changes was also minor in the case of HAS interaction with DOX or PTX and β-lactoglobulin interaction with DOX, in line with our results^15,37,41^.

**Figure 4:**
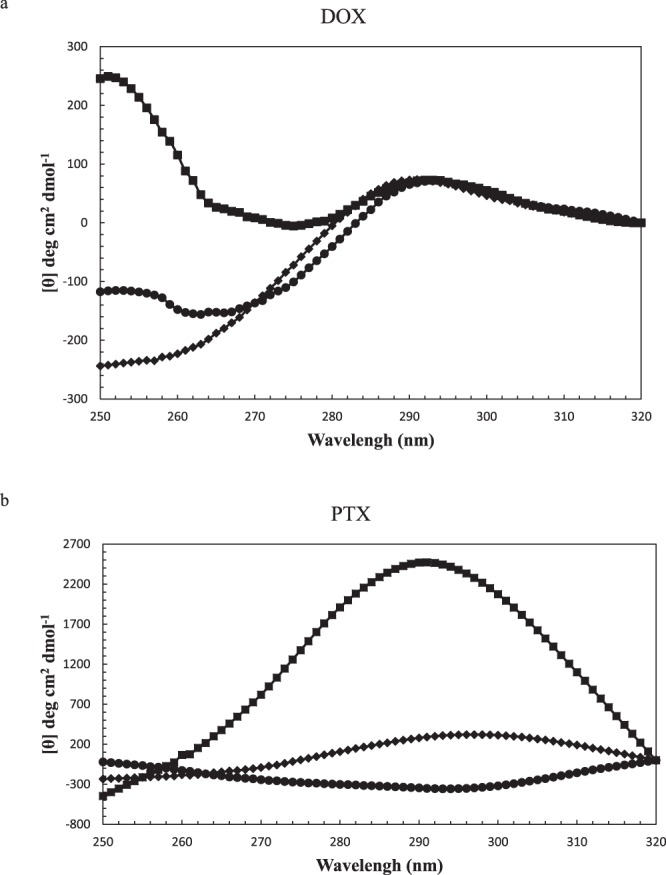
Near-UV CD spectra of α-Lac in the absence and present of (**a**) DOX and (**b**) PTX. 10 µM α-Lac dissolved in Tris buffer (20 mM, pH 7) was scanned in the absence (♦) and presence of 2 (●) and 5.5 (■) stochiometries of DOX or PTX at room temperature (25 °C).

The near-UV CD spectra of 1:2 and 1:5.5 ratios of α-Lac:DOX and α-Lac:PTX complexes, shown in Fig. 4a,b, reflected conformational transitions. α-Lac:DOX systems presented more positive ellipticity in 260–280 nm, which may reflect perturbed environments of aromatic amino acids except for TRP^42^. This indicates less or no participation of TRP in the interaction of DOX with α-Lac, which agrees with our simulation studies performed by docking. In fact, according to the docking studies, DOX does not interact with α-Lac’s TRPs. However, α-Lac:PTX systems showed changes in 295 nm, which indicates greater participation of TRP. Accordingly, our docking simulation studies showed greater involvement of TRP 60 during interaction of PTX with α-Lac. Interestingly, the 1:2 ratio of α-Lac:PTX caused negative ellipticity in 295 nm while the 1:5.5 ratio of α-Lac:PTX resulted in a highly positive ellipticity in the same wavelength. As a matter of fact, in the presence of 20 µM PTX, the chemical nature of TRP microenvironment is such that left-handed circularly polarized light (L-CPL) is absorbed to a lesser extent than right-handed circularly polarized light (R-CPL), which leads to appearance of a negative peak in 295 nm. However, higher concentrations of PTX, which causes higher absorbance of L-CPL compared to R-CPL brings about the positive peak^43^.

In the case of DOX, more intense ellipticity in 260 nm could be attributed to an electron transfer between oxygens containing DOX and the TYR of α-Lac^42^. Moreover, when there is TYR- TRP sequence of amino acids, a weak peak at 295 nm is observed which could be seen in the case of DOX and 20 µM PTX^44^. However, higher concentrations of PTX provided huge changes in 295 nm possibly due to generation of another binding site in the vicinity of TRP 60, which was confirmed by our computer simulations.

### Pace analysis to determine protein thermal stability

**Figure 5:**
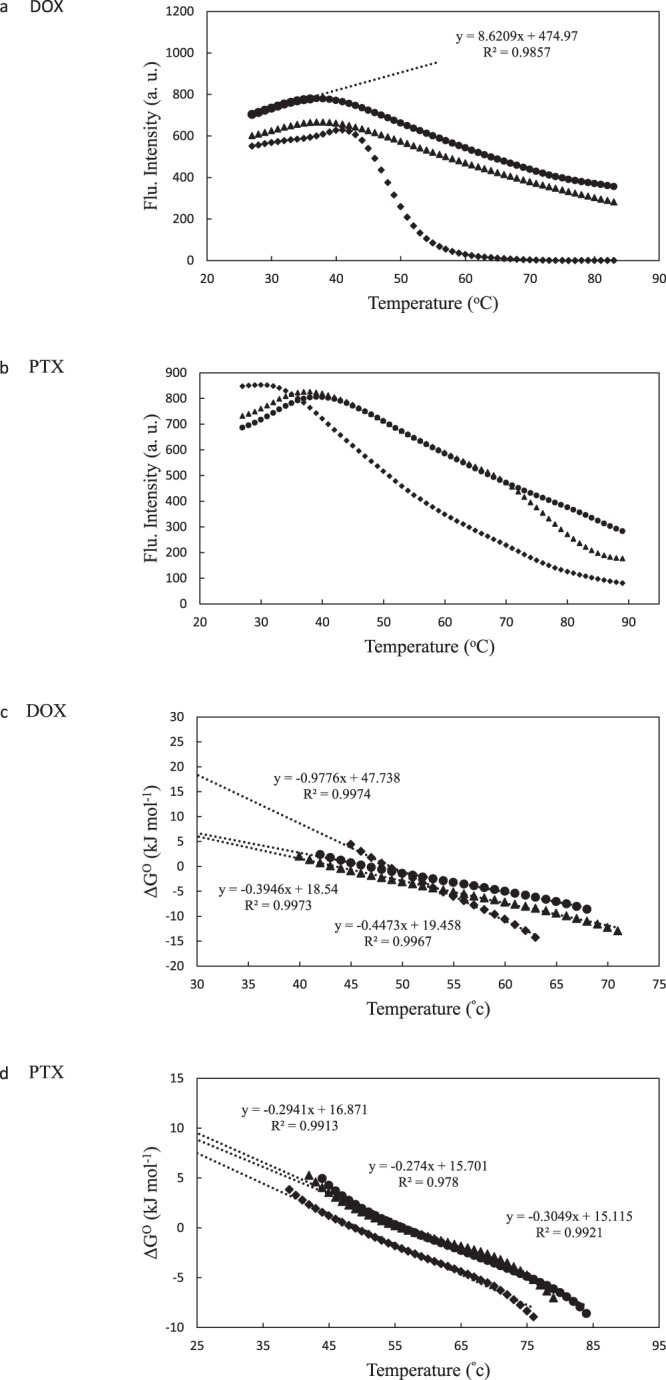
Pace analysis diagrams to determine protein thermal stability. Heat denaturation profiles (**a** and **b**) and standard Gibbs energy of unfolding plots (**c** and **d**) of 10 mM α-Lac in 20 mM Tris buffer (pH 7) were determined in the absence and presence of various concentrations of DOX or PTX. Excitation and emission wavelengths were 280 and 337 nm, respectively. α-Lac (●), α-Lac:DOX or α-Lac:PTX ratio of 1:2 (▲), and α-Lac:DOX or α-Lac:PTX ratio of 1:5.5 (♦).

Thermal stability of α-Lac upon interaction with DOX or PTX was studied by fluorescence spectroscopy. The changes in fluorescence intensity of α-Lac at 337 nm *versus* temperature in the presence and absence of various concentrations of DOX or PTX is shown in Fig. 5a–d, respectively. Increasing fluorescence intensity in the temperature range of 25−40 °C reflects the decrease of intramolecular quenching of TRP fluorescence^45^. Denatured fraction of α-Lac (*f*_d_) can be calculated according to equation (1) using Fig. 5a,b ^46^:

1 $${f}_{d}=\frac{{F}_{n}-{F}_{obs.}}{{F}_{n}-{F}_{d}}$$

where F_obs._ represents the observed fluorescence intensity of α-Lac at 337 nm; F_n_ and F_d_ represent fluorescence intensities of native and denatured states, respectively. As can be seen in the Fig. 5a,c, the initial points represent the native state of α-Lac for which the equation of the line was determined. Accordingly, F_n_ was calculated for each α-Lac:DOX and α-Lac:PTX ratio. The fluorescence intensity at 95 °C was considered as the denatured state (F_d_). As a first approximation, protein denaturation can be approached using the two state theory (Native ↔ Denatured), and the equilibrium constant (*K*) value was calculated by equation (2) ^46^:

2 $$K=\frac{{f}_{d}}{1-{f}_{d}}$$

Furthermore, using equations (3) and (4), the value of change in standard Gibbs free energy (*∆G*°) for denatured α-Lac was determined^46^:

3 $$\Delta G^\circ =-\,RT\,\mathrm{ln}\,K$$

4 $$\Delta G^\circ =-RT\,\mathrm{ln}(\frac{Fn-{F}_{obs.}}{{F}_{obs.}-Fd})$$

Furthermore, *∆G°* as a function of temperature is plotted in Fig. 5b,d, which depicts *∆G°* linear variation with temperature over the unfolding region. In order to estimate the conformational stability at room temperature (*∆G°*_25_), it is necessary to assume that a linear dependence is valid until 25 °C.

According to the results presented in Table 3, it was revealed that lower concentrations of DOX or PTX caused minor changes in the α-Lac stability while higher concentrations led to significant changes in thermal stability of the protein. Interestingly, DOX or PTX showed different effects; 5.5 fold stoichiometric excess of DOX induced significantly higher thermal stability of α-Lac whereas PTX caused marked decrease in α-Lac thermal stability. We attributed this to the conformational changes due to generation of another binding site in α-Lac. The results of Pace analysis are in a good agreement with DSC findings.

**Table 3:** DOX or PTX binding to α-Lac by Pace analysis.

Sample	*T*_*m*_ (^o^C)	*ΔG*^*O*^ (kJ mol^-1^)
α-Lac (Control)	38	8.4
α-Lac:DOX (1:2)	38	8.3
α-Lac:DOX (1:5.5)	41	23.3
α-La (Control)	39	9.5
α-Lac:PTX (1:2)	38	8.9
α-Lac:PTX (1:5.5)	31	7.5

### Lifetime measurements

Based on the results of fluorescence lifetime measurements, τ_1_ of 0.91 and τ_2_ of 3.53 ns with amplitudes of 61.76 and 38.24%, respectively, were obtained for α-Lac. Therefore, the average τ of 1.91 ns was calculated according to the equation (7) in the “Experimental and Theoretical Methods” section (χ^2^ = 1.273). Furthermore, adding 0, 2.0, and 5.5 μM DOX or PTX to the α-Lac solution did not cause significant changes in the fluorescence lifetime. This implies that the interactions between α-Lac and DOX or PTX are of static nature which means that DOX or PTX bound to α-Lac dissociate from their sites in hydrophobic pockets after a definite time while free DOX or PTX in the solution replace them. This already has been shown in the case of HAS and PTX in which the changes in fluorescence lifetime was slight and the interaction was statistic. The reported results are in line with our findings^47^. As it will be seen, this finding would be of importance in our cytotoxic studies.

### Anisotropy

**Figure 6:**
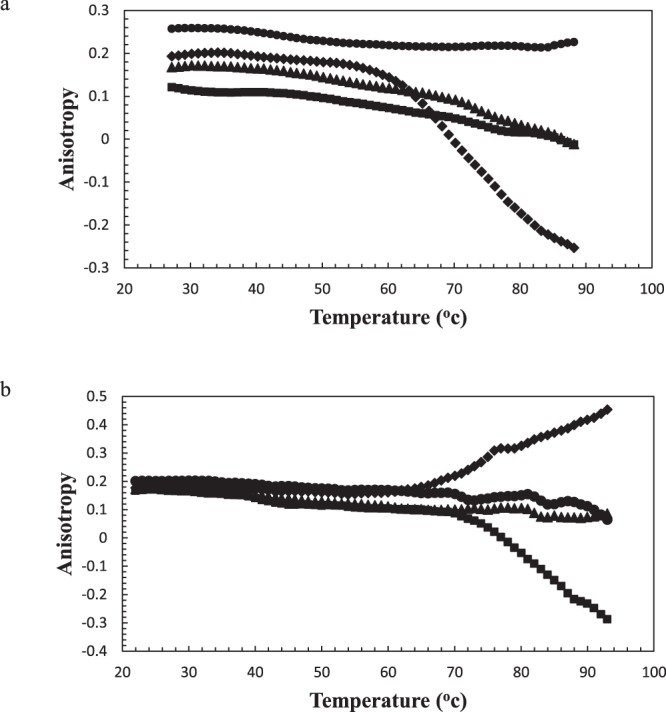
Anisotropy of α-Lac in the presence of (a) DOX and (b) PTX. Anisotropy of 10 µM FITC-labeled α-Lac against temperature was obtained in absence (●) and presence of 10 (♦), 20 (■), and 40 (▲) µM DOX or PTX. The protein was dissolved in 20 mM Tris buffer (pH 7).

α-Lac was labeled with FITC and the anisotropy of the labeled protein was measured before and after DOX or PTX binding. The dye to protein ratio was low and the anisotropy of unlabeled protein was 0.26. The results obtained from anisotropy measurements are displayed in Fig. 6a,b. According to the results, addition of DOX or PTX to α-Lac solution reduced the anisotropy value to 0.12 and 0.17, respectively. Such a decrease in anisotropy might be caused by incident of homo-FRET between FITC molecules and may imply formation of protein assemblies. If labelled proteins associate, fluorophores get closer together and excitation energy transfers amongst them and their emission becomes unpolarized^48^. In the presence of hydrophobic ligands, proteins may form protein assemblies^29,49^ and in such case FITC molecules may become close enough for the resonance energy transmission to happen.

As it can be seen in Fig. 6a,b, in 1:1 ratio of α-Lac:PTX, the proteins are not saturated yet. In fact, raised temperature caused free proteins to bind preliminary α-Lac:PTX complex9es and resulted in larger protein assemblies and consequently decreased anisotropy. This was also observed in the case of 1:1 stoichiometry of α-Lac:DOX complexes. However, in 1:2 ratio of α-Lac:PTX, smaller and sphere-shaped protein assemblies were formed due to increased temperature. This means unavailability of the α-Lac hydrophobic patches for the solvent in lower concentrations of PTX, which resulted in diminished hydrophobic interactions between protein molecules and subsequent dissociation of α-Lac:PTX complexes. This could be attributed to conformational changes in α-Lac and generation of another binding site upon interaction with PTX, which remains in agreement with our CD and DSC findings. Although this is not the case for DOX, which agrees with structural studies. Finally, at 1:4 stoichiometry of α-Lac:DOX or α-Lac:PTX, the proteins were approaching saturation and so the formed complexes due to raised temperature were preserved.

### Molecular docking

**Figure 7:**
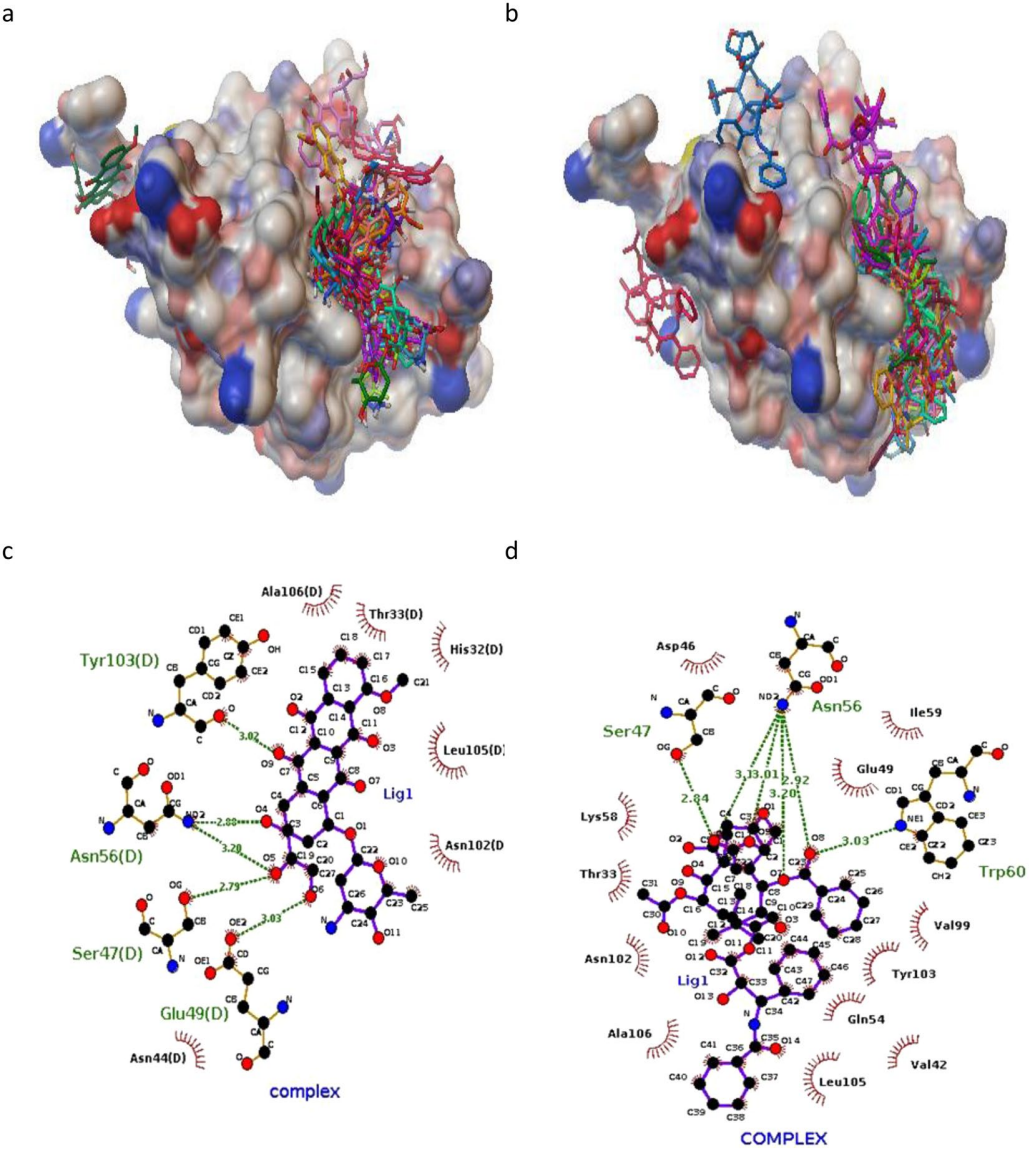
Binding sites and residues interacted. Possible binding sites of α-Lac for (**a**) DOX or (**b**) PTX; LIGPLOT diagrams of the interacted residues between a-La and (**c**) DOX or (**d**) PTX.

Blind and local docking were performed to indicate possible binding sites for ligands in the protein. According to the blind docking results, two and three binding sites on α-Lac were predicted for DOX and PTX, respectively (Fig. 7a,b). As presented in Fig. 7c,d, the best position for DOX and PTX was located in the same hydrophobic pocket close to antiparallel β sheet of α-Lac. According to the AutoDock Vina calculations, binding affinities of -7.1 and -8.0 (kcal/mol) were obtained for α-Lac:DOX and α-Lac:PTX, respectively. Additionally, local docking was performed based on the results of blind docking. Besides, analysis of the docking results revealed the residues within a distance cutoff of 3.5 Å from DOX: ASN 44, SER 47, GLU 49, ASN 56, TYR 103, LEU 105, ALA 106. However, HIS 32, ASP 46, SER 47, GLU 49, ASN 56, LYS 58, TRP 60, ASN 102, TYR 103, LEU 105 were detected to locate in 3.5 Å from PTX. In both cases, docking results for binding sites and binding affinities are in a good agreement with experimental data obtained using ITC.

Based on the results of the modeling, dominant interaction is hydrophobic and hydrogen bond. As indicated in Fig. 7c,d, DOX interacted with SER47, GLU 49, ASN 56, and TYR 103 and PTX interacted with SER47, ASN 56, and TRP 60 via hydrogen bonding in the hydrophobic pocket of α-Lac. Other residues are shown to have hydrophobic interactions with α-Lac.

### Molecular dynamics simulation

**Figure 8:**
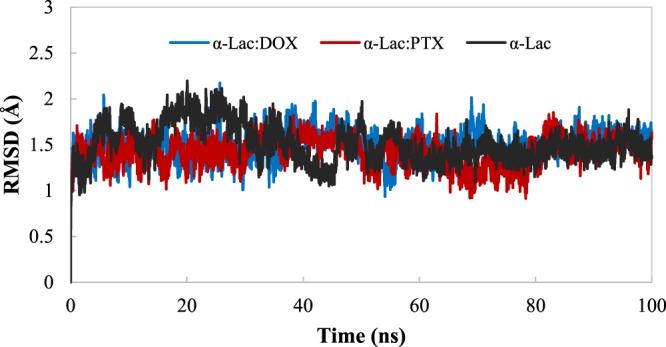
RMSD of α-Lac backbone relative to the initial structure. RMSD of α-Lac in the absence or presence of DOX or PTX during 100 ns MD simulation was calculated.

Three MD simulations were carried out for α-Lac and α-Lac:DOX and α-Lac:PTX complexes. Root mean squared deviation (RMSD), solvent accessible surface area (SASA) and the secondary structure (with DSSP algorithm) were studied to analyze the structures^50^. Generally, the time evolution of RMSD shows the simulation stability. Furthermore, it is considered as a way to evaluate the results of molecular docking. Moreover, RMSD of the protein backbone was calculated relative to the initial structure in the presence and absence of DOX or PTX molecules (Fig. 8). Based on the results, conformation of the α-Lac in the presence or absence of DOX or PTX reached stability, indicating that all of structures (obtained from local docking) were fully stable. Upon binding to DOX or PTX, the RMSD values for α-Lac were decreased (average values of 1.51, 1.46, and 1.42 Å for α-Lac, α-Lac:DOX, and α-Lac:PTX, respectively). The reduction in RMSD for α-Lac:PTX relative to α-Lac was higher than that for α-Lac:DOX, suggesting the greater stability of α-Lac:PTX complexes according to the experimental data obtained using ITC.

**Figure 9:**
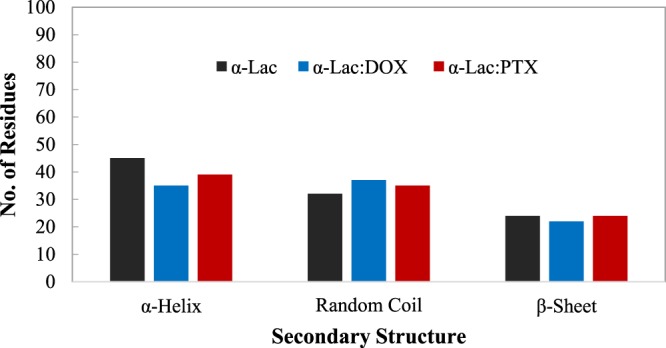
Analysis of secondary structure. Secondary structure was analyzed using DSSP program. Number of residues adopting α-Helix, random coil, and β-Sheet secondary structures was determined.

SASA is another parameter for investigating the conformational changes of the protein structure which calculated in these simulations. The average values of SASA are 7137, 7162, and 7175 Å2 for α-Lac, α-Lac:DOX, and α-Lac:PTX, respectively. These data show the greater exposure of α-Lac surface to solvent in the presence of DOX and PTX molecules which are agreement with experimental data. Furthermore, secondary structure was analyzed using DSSP program. As shown in Fig. 9, the number of residues adopting secondary structural elements was calculated. According to the results, α-helix content decreases in α-Lac, upon binding to DOX or PTX (45, 35, and 39% for α-Lac, α-Lac:DOX, and α-Lac:PTX, respectively). Besides, binding of DOX or PTX to α-Lac resulted in an increased random coil content (32, 37, and 35% for free α-Lac, α-Lac:DOX, and α-Lac:PTX, respectively). Other secondary structural elements especially β-sheets did not present any significant alteration. These clearly are supported by our far-UV CD results.

**Figure 10:**
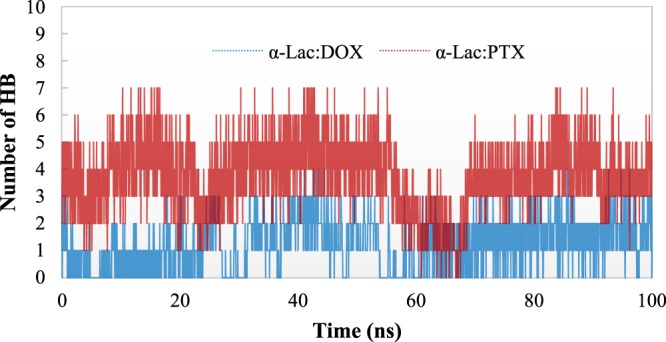
Number of Hydrogen Bonds. Number of Hydrogen Bonds during the MD simulations for α-Lac:DOX and α-Lac:PTX complexes.

The hydrogen bond analysis was performed to study the interactions in α-Lac:DOX and α-Lac:PTX complexes (Fig. 10). In the hydrogen bond analysis, a hydrogen bond was defined as a (dDA) <3.5 Å distance and a (αDHA)> 135° angle between the hydrogen bond donor and acceptor. The data obtained from the hydrogen bond analysis indicated that the number of hydrogen bonds in α-Lac: PTX complex is more than those of α-Lac:DOX complex. These finding can be attributed to the orientation of the hydrogen bond donors and acceptors in PTX which is more accessible than relative to DOX.

The stability of hydrogen bonds was measured by % occupancy (Table 4). Our findings show the stability of hydrogen bonds in α-Lac:PTX complex was higher than those of α-Lac:DOX complex. These data were consistent with the analysis discussed in the other sections.

**Table 4:** Hydrogen bond analysis of α-Lac:DOX and α-Lac:PTX complexes during the MD simulation.

α-Lac:DOX		α-Lac:PTX	
Residues	% Occupancy	Residues	% Occupancy
THR 33	15.9	ASP 46	52.6
SER 47	33.8	GLU 49	44.2
GLN 54	37.4	ASN 56	36.5
ASN 56	29.6	LYS 58	49.8
LYS 58	39.1	TRP 60	24.5
TYR 103	44.9	ASN 102	75.5
		TYR 103	27.3

**Figure 11:**
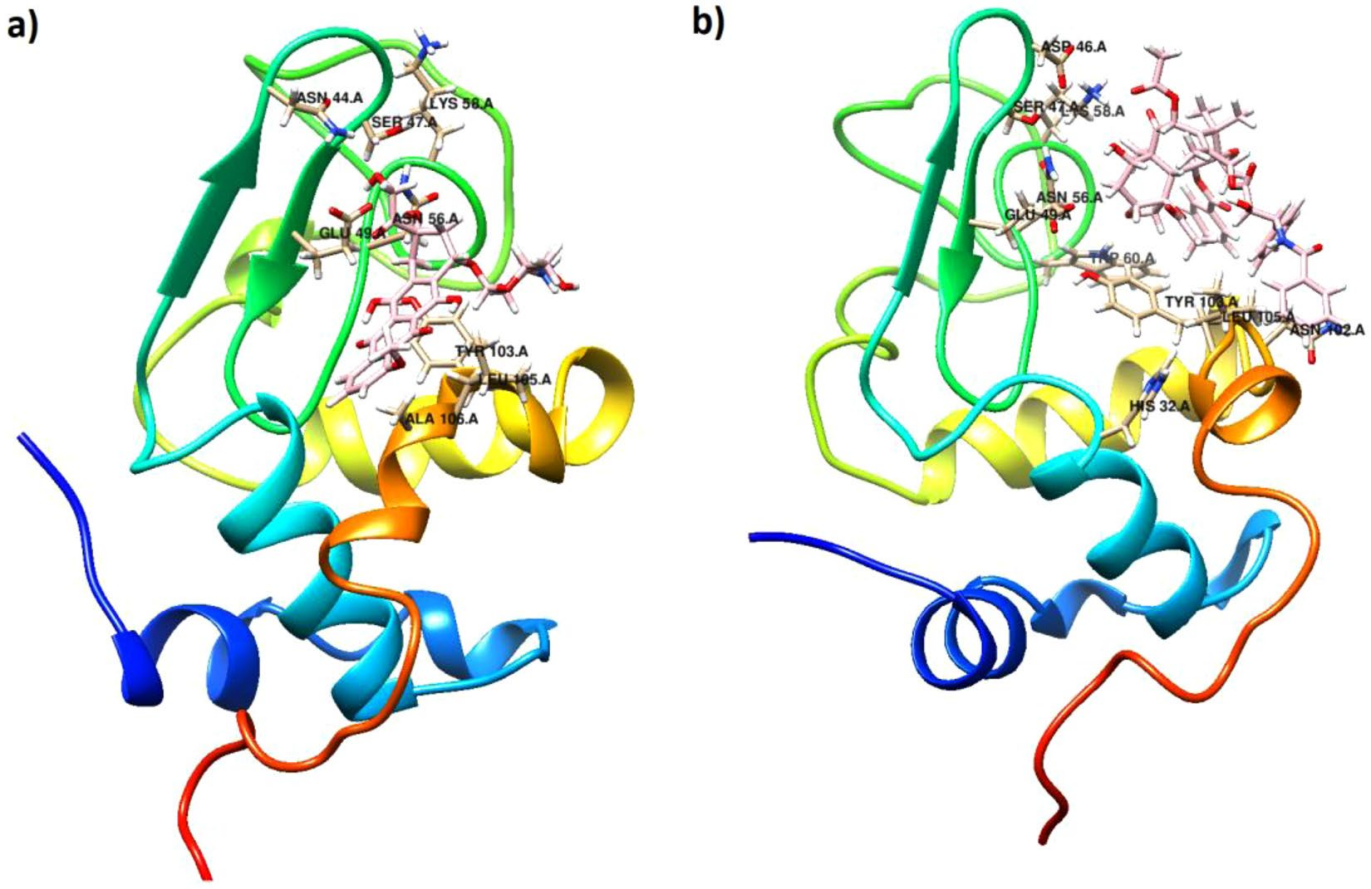
Conformation of the average structure of (**a**) the α-Lac:DOX and (**b**) α-Lac:PTX complexes for the last 20 ns of the MD simulation. DOX, PTX, and interacting residues of α-Lac are shown in the stick representations. The α-Lac structure is depicted in cartoon representations (Color scheme for α-Lac: cyan for carbon, red for oxygen, blue for nitrogen, and white for hydrogen atoms. For DOX, PTX, carbon atoms are in pink).

Furthermore, the average structures for the last 20 ns of the MD simulation for α-Lac:DOX and α-Lac:PTX complexes were investigated and are presented in Fig. 11a,b. During the MD simulation, both DOX and PTX were positioned in the same hydrophobic pocket close to antiparallel beta sheet of α-Lac. The ligand molecules oriented in such a way that they had the greatest interactions with the α-Lac residues. Furthermore, the interacting residues of α-Lac are depicted in the Fig. 11a,b. These data agree with results obtained from molecular docking.

### Cell proliferation assay

**Figure 12:**
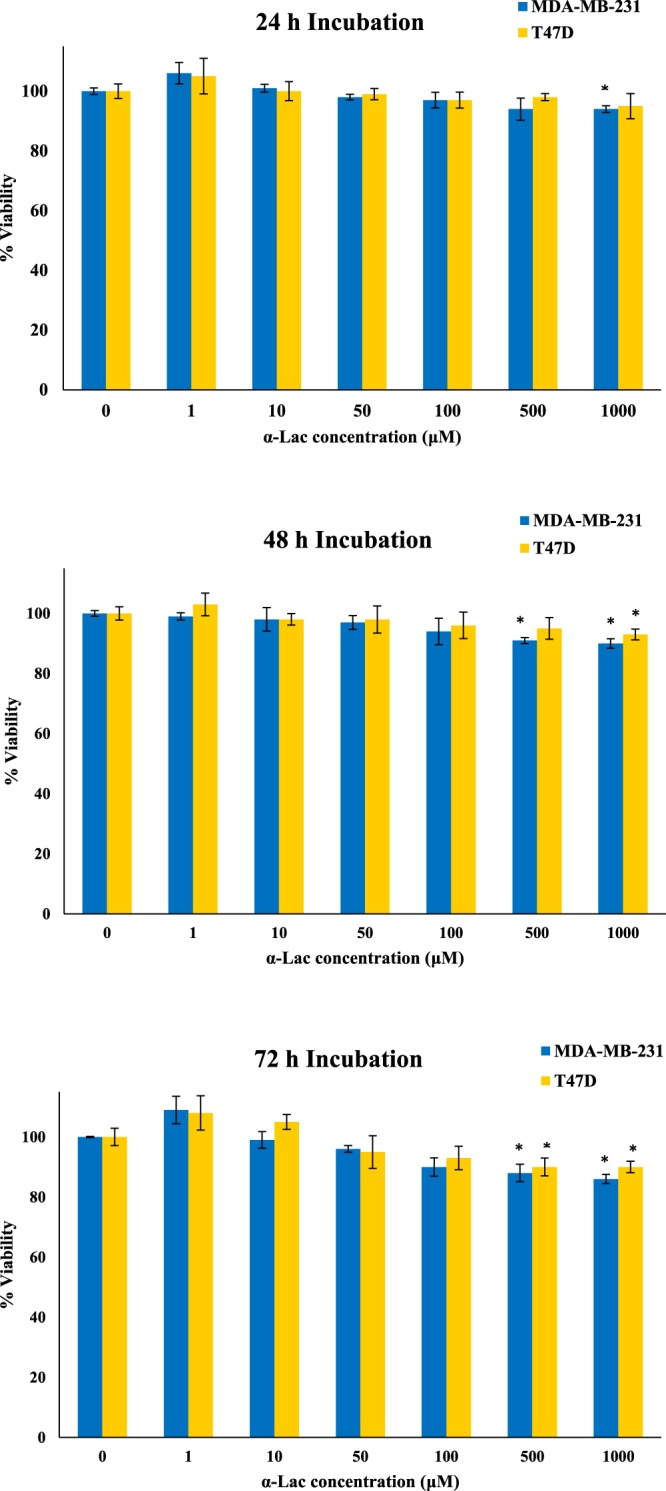
Cytotoxicity of α-Lac in MDA-MB-231 and T47D cells. The cells were incubated with 1–1000 μM of α-Lac for 24 (**a**), 48 (**b**), and 72 (**c**) h. Cell viability was evaluated by MTT assay. The data represents mean ± SEM of three independent experiments. *represents p < 0.05 compared to the corresponding control.

**Figure 13:**
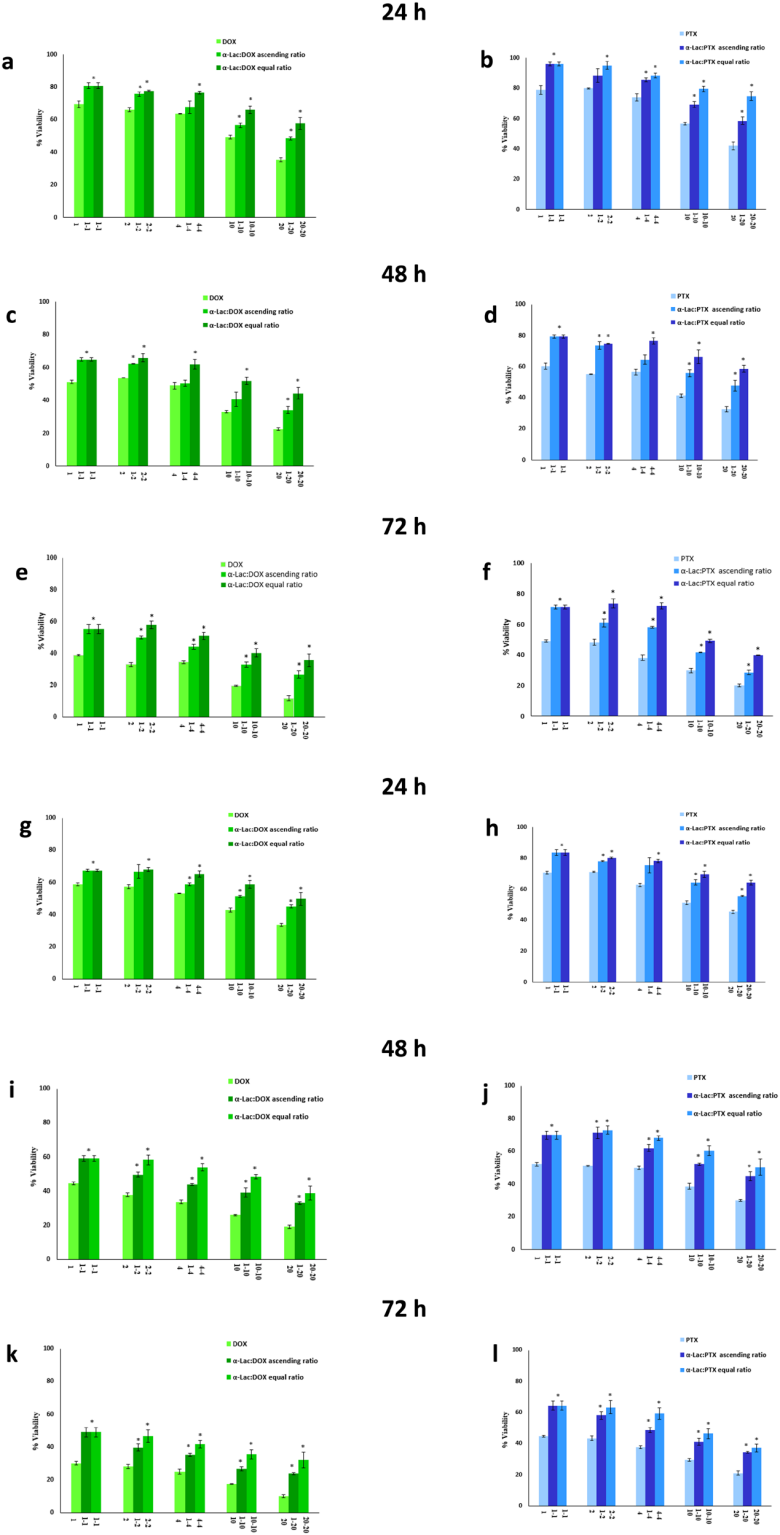
MTT assay. Effect of DOX alone (1, 2, 4, 10, and 20 μM), PTX alone (1, 2, 4, 10, and 20 μM), and α-Lac (1, 2, 4, 10, and 20 μM) along with DOX or PTX (1, 2, 4, 10, and 20 μM) with equal (1:1, 2:2, 4:4, 10:10, and 20:20) and ascending ratios (1:2, 1:4, 1:10, and 1:20) in MDA-MB-231 (**a–f**) and T47D (**g–l**) cells. The cells were incubated with the described concentrations and ratios of α-Lac, DOX, and PTX for 24 (**a,b,g** and **h**), 48 (**c,d,i** and **j**), and 72 (**e,f,k** and **l**) (**h)**. Cell viability was evaluated by MTT assay. The percent viability data were normalized to the corresponding control. The data represents an average ± SEM of three independent experiments. *represents p < 0.05 compared to the corresponding control.

The cytotoxicity of α-Lac:DOX or α-Lac:PTX was compared with that of the free α-Lac, DOX, and PTX against MDA-MB-231 and T47D cells following 24, 48, and 72 h incubation using MTT assay. Fig. 12a–c presents the cytotoxicity of α-Lac in the extensive range of 1-1000 μM on MDA-MB-231 and T47D cells. Based on our results, α-Lac could only induce statistically significant toxicity at higher concentrations of 500 and 1000 μM. It is worth mentioning that, α-Lac elicited insignificant cytotoxicity at the concentrations used in complex with DOX or PTX. Moreover, free DOX or PTX decreased viability of MDA-MB-231 and T47D cells in a time- and concentration-dependent manner (Fig. 13a–l). The data in Fig. 13a–l were normalized to the corresponding control (MDA-MB-231 and T47D cells treated with 1, 2, 4, 10, and 20 μM DOX or PTX). According to our results, DOX showed higher cytotoxicity compared to PTX in both cell lines and at the three incubation times, which have been reported by other authors^51^. Furthermore, based on our MTT results, the presence of α-Lac could decrease the cytotoxicity of DOX or PTX whether applied in equal or ascending ratios at 24, 48, and 72 h. This could stem from the interactions of DOX or PTX with α-Lac and subsequent reduction of free drug concentrations. A related point to consider is that these interactions are stable until 72 h and could decrease DOX or PTX toxicity up to 72 h which could be an indication of static interaction of α-Lac with DOX or PTX. This reflects the fact that some drug molecules present in the culture media were free and part of them bound to α-Lac molecules after the interactions between α-Lac and DOX or PTX reached their equilibria. In fact, there was an equilibrium between α-Lac and free drugs present in the solution, i.e. over time, part of bound drugs dissociate and enter the culture media and part of free drugs replace them in turn. Consequently, free effective DOX or PTX concentrations could be lower in the presence of α-Lac than its absence. Over a time, the free drugs entered the cells and caused their death, which means their concentrations in the culture media has decreased. Therefore, in order to reach an equilibrium again, less drugs were bound to the protein. This could indicate slow release of the drugs which is considered as an important principle in drug delivery. Slow release of drugs could result in less drug injections and consequently less damage to healthy tissues which is very desirable.

Besides, α-Lac was more effective in toxicity reduction of DOX or PTX when applied in equimolar ratio. This could be due to the fact that there are enough α-Lac molecules available to interact with DOX or PTX when applied in equal ratio while less α-Lac molecules are present when DOX or PTX were applied at ascending ratio. Thus, additional free DOX or PTX molecules are available to penetrate the cells when applied ascending stoichiometries. Interestingly, α-Lac was more efficient in reducing PTX toxicity than DOX, which is in line with our thermodynamic results. As reported in thermodynamics section, since there are two binding sites for PTX on α-Lac and α-Lac:PTX’s K_b_ is higher than that of α-Lac:DOX, it can be concluded that more PTX molecules interacted with α-Lac compared with DOX. In other words, free PTX concentration is smaller in α-Lac:PTX than free DOX concentration in α-Lac:DOX solutions.

### Apoptosis assay

**Figure 14:**
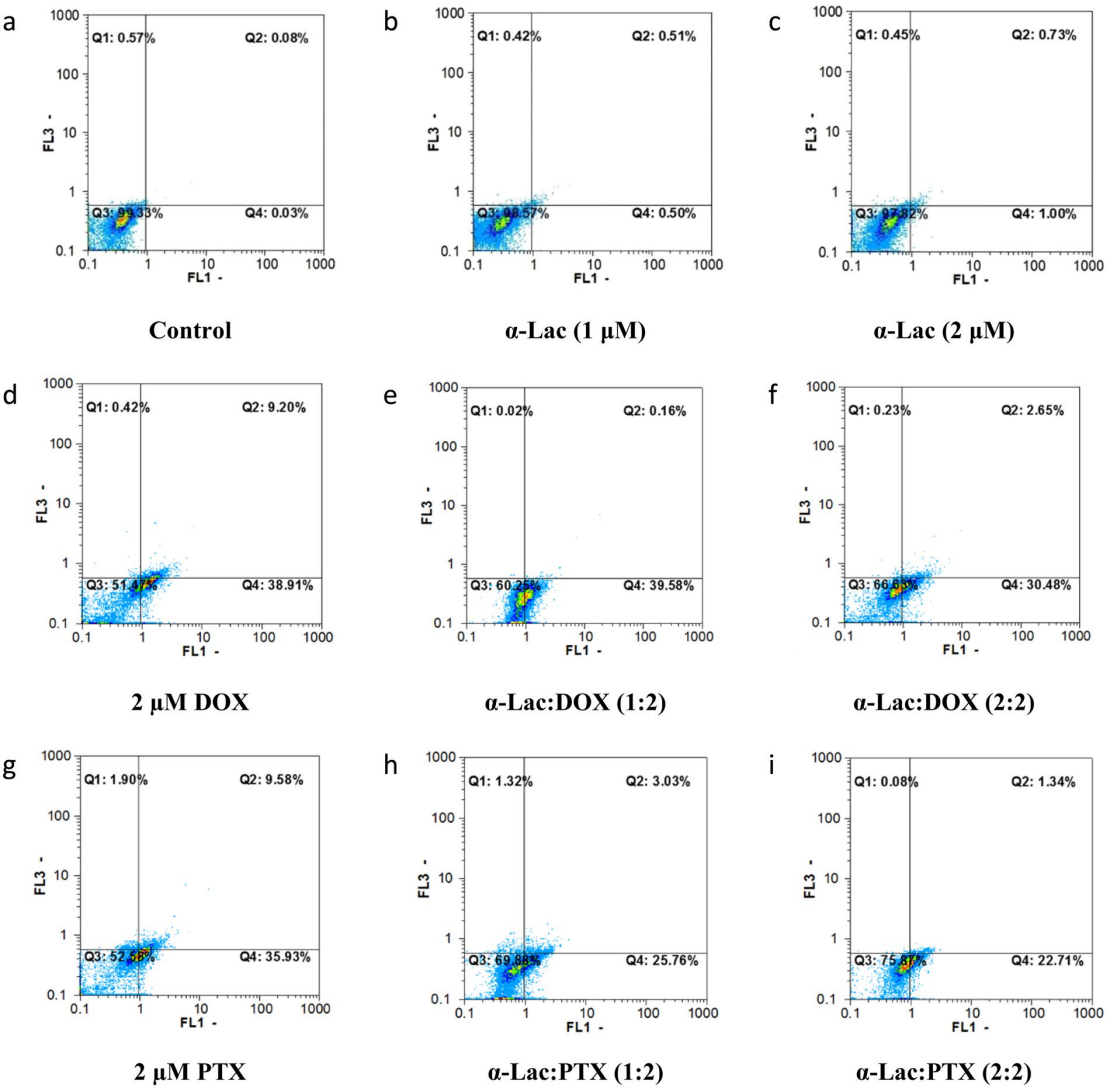
Flow cytometric analysis of apoptosis induction. Effect of α-Lac, DOX, PTX, α-Lac:DOX, and α-Lac:PTX on apoptosis induction in MDA-MB-231 cells after incubation for 48 h. The cells were incubated with 1 (**b**) and 2 (**c**) μM α-Lac, 2 µM DOX (**d**), 1:2 ratio of α-Lac:DOX (**e**), 2:2 ratio of α-Lac:DOX (**f**), 2 μM PTX (**g**), 1:2 ratio of α-Lac:PTX (**h**), and 2:2 ratio of α-Lac:PTX (**i**). The presented graphs are representative of the flow cytometric output. The control cells were also presented to compare (**a**).

**Figure 15:**
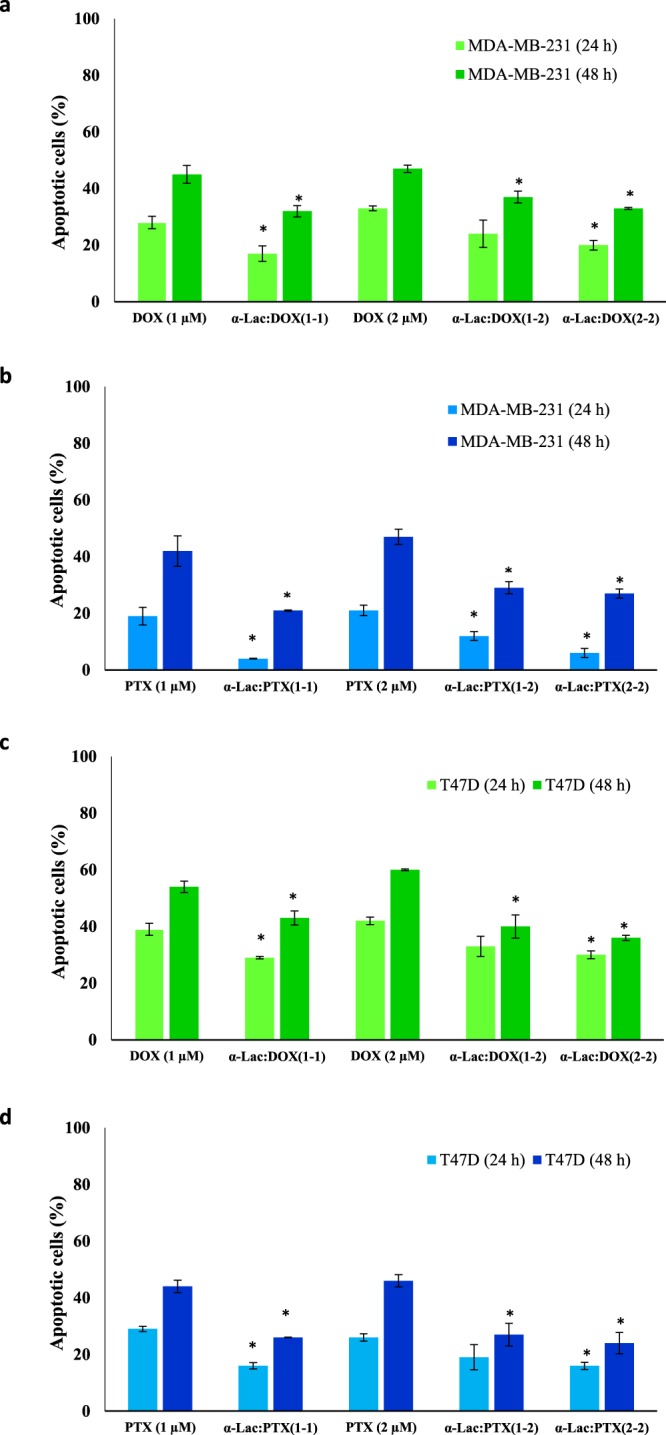
Quantification of apoptosis analysis using flow cytometry. Effect of α-Lac on apoptosis induction by DOX or PTX when applied in equal or ascending ratios in MDA-MB-231 (**a**,**b**) and T47D (**c**,**d**) cells. The cells were incubated with 1 and 2 μM DOX or PTX alone or in complex with 1 and 2 μM α-Lac in equal (1:1 and 2:2) or ascending (1:2) ratios for 24 and 48 h. Apoptosis induction was evaluated by flow cytometry. Each data point is an average ± SEM of three independent experiments. *represents p <0.05 compared to the corresponding control.

Apoptosis induction by α-Lac alone, DOX alone, PTX alone, and α-Lac:DOX or α-Lac:PTX complexes were evaluated by flow cytometry. In the current study, different staining of viable and apoptotic cells by AO and EB were applied to assess apoptosis by flow cytometry. Representative flow cytometric outputs of treated MDA-MB-231 cells after 48 h incubation are displayed in Fig. 14a–i. FL1-H and FL3-H channels of the flow cytometer were used to measure green (AO) and red (EB) fluorescence intensities, respectively. Q1 and Q2 regions were corresponding to necrotic and late apoptotic cells whereas Q3 and Q4 regions represented viable and early apoptotic cells, respectively. According to the results (Fig. 15a–d), treatment with DOX or PTX could induce total apoptosis (sum of early and late apoptosis) in MDA-MB-231 and T47D cells in a time- and concentration-dependent manner. It should be noted that the data in Fig. 15a–d were normalized to the corresponding control (MDA-MB-231 and T47D cells treated with 1 and 2 μM DOX or PTX). Interestingly, presence of α-Lac caused reduction of the apoptosis induced by DOX or PTX either applied in equal ratios with DOX or PTX or in ascending ratios in both cell lines after 24 and 48 h treatment. However, equal ratios of α-Lac and DOX or PTX resulted in reduced amounts of apoptosis induction than ascending ratios in MDA-MB-231 and T47D cells in 24 and 48 h. A related point to consider is that, 1 and 2 μM α-Lac alone did not provoke apoptosis induction in MDA-MB-231 and T47D cells after 24 and 48 h incubation (Fig. 2, Supplementary information). Clearly, the results obtained from apoptosis and MTT assays were highly consistent in our study. Thus, it was concluded that α-Lac could be introduced as a suitable carrier for chemotherapeutic agents DOX or PTX.

## Conclusion

In the current study, it was suggested that α-Lac may serve as a novel and appropriate carrier for chemotherapeutic agents DOX or PTX as supported by thermodynamic, spectroscopic, computational, and cellular studies. Based on our studies, van der Waals forces and hydrogen bonds were shown to be involved in the interactions between ligands and α-Lac. Furthermore, it was revealed that the interactions between α-Lac and DOX or PTX are of static and spontaneous nature. According to the results, one binding site for DOX and two binding sites for PTX were detected. Based on spectroscopic and computational studies, it was revealed that TRP 60 of α-Lac is engaged in interaction with PTX. While, no TRP is involved in α-Lac’s interaction with DOX. In addition, α-Lac interaction with DOX or PTX led to greater exposure of α-Lac hydrophobic patches. Consequently, protein-protein interactions were facilitated by α-Lac:ligand interactions. Besides, DOX caused higher thermal stability of α-Lac while PTX resulted in its lower thermal stability. Interestingly, α-Lac could lower the cytotoxicity of DOX or PTX as revealed by MTT and apoptosis assays. It seemed that, α-Lac interactions with DOX or PTX could result in their slower release that is desirable in drug delivery. Since the interaction of α-Lac with DOX or PTX was static in nature, the formed complexes were stable up to at least 72 h. However, α-Lac presented more potential in reducing PTX toxicity compared to DOX as revealed by cytotoxicity studies and confirmed by thermodynamic findings.

## Materials and Methods

### Materials

α-Lac from bovine milk-type III-calcium depleted (L 6010), (3-(4,5-dimethylthiazol-2-yl)-2,5-dephenyltetrazolium bromide (MTT), dialysis membranes (cut-off, 12000 Da), acridine orange, Ludox®, penicillin, and tris(hydroxymethyl) aminomethane (Tris) were obtained from Sigma-Aldrich. Fetal bovine serum (FBS), RPMI 1640 culture medium, and trypsin were purchased from GIBCO. DOX-HCl, ethidium bromide, ethylenediaminetetraacetic acid (EDTA), and dimethyl sulfoxide (DMSO) were from Merck. Fluorescein 5-isothiocyanate (FITC) and PTX were obtained from Thermo-Fisher Scientific (Waltham, MA) and Stragen Pharma, respectively. Flasks, petri dishes, and 96-well plates were purchased from Nunc (Denmark).

### Cell lines and culture conditions

MDA-MB-231 and T47D breast cancer cell lines were received from National Cell Bank of Iran (Pasteur Institute, Iran). Both cell lines were cultured in RPMI 1640 medium supplemented with 10% heat-inactivated fetal bovine serum (FBS), 500 μg/ml penicillin, and 200 μg/ml streptomycin. The cells were incubated in a humidified atmosphere (95%) with 5% CO_2_ at 37 °C. MDA-MB-231 and T47D cells were routinely sub-cultured using trypsin/EDTA in phosphate buffered saline (PBS) solution^52^.

### Experimental setup

In the current study, 20 mM Tris buffer, the most common buffer used for α-Lac, containing 3.5 mM EDTA to remove the bound Ca^2+^, was applied during the thermodynamic, spectroscopic, and cellular experiments as a solvent for α-Lac. Furthermore, ethanol was used to dissolve PTX in all the experiments. It is important to note that, the amount of ethanol used in all the experiments was less than 3% (v/v) which is lower than maximum 20% (v/v) capable of eliciting conformational changes in the α-Lac, as reported in the published literature^53,54^. Besides, the authors are willing to emphasize that the effect of the amount of 3% (v/v) ethanol was measured on α-Lac structure in isothermal titration and differential scanning calorimetries and detected no change which has been documented in our previous publication^31^. In addition, it is necessary to emphasize that during all the spectroscopic, thermodynamic, and cellular experiments, the protein was soluble and never precipitated after forming complexes (*vide infra*) with DOX or PTX.

In the case of thermodynamic and spectroscopic studies, it should be taken into account that, since there is a decrease of 0.025 unit in pH of Tris buffer for 1 °C of temperature raise (according to the protocol released by the manufacturer, Sigma-Aldrich), the total amount of changes in pH during temperature raise from 25 to 90 °C would be nearly 1.5 unit (pH 5.5). However, according to the published literature, α-Lac is resistant to pH fluctuations up to pH 4^55^. Moreover, in order to exclude the probable pH changes due to titration with DOX or PTX solutions, i.e. the highest amount was 15% and 6% (v/v) of the total volume at the end of titrations, respectively, the authors measured the pH and detected no change.

### Isothermal titration calorimetry

Heat flow was measured using isothermal titration calorimetry (ITC) to determine type of the interactions. ITC assays were performed on a VP-ITC Microcalorimeter (MicroCal, LLC, Northampton, MA, USA) system at 25 °C. The 290 µM α-Lac, loaded in the sample cell, was titrated with 3.4 mM DOX while 50 µM α-Lac was titrated with 250 µM PTX in pH 7.0. In the both cases, the initial volume of α-Lac was 1.8 ml. The reference cell was loaded with 20 mM Tris buffer (pH 7.0). In each experiment, all the solutions were degassed under vacuum for 3 min before loading in the ITC cells. The measurements were started by injecting 5 μl of the titrant into the reactor, which were proceeded by 29 successive injections. These injections were performed at a rate of 10 µl per 20 s with 3 min spacing time between them. To correct the heat effects due to mixing and dilution, the heat effects due to PTX and DOX in protein buffer and the effect of ethanol as PTX’s solvent under identical condition (temperature and timing) were subtracted from the heat of α-Lac:DOX and α-Lac:PTX titrations. The experimental data were analyzed and fitted using MicroCal Origin software to calculate the binding parameters.

### Differential scanning calorimetry

Differential scanning calorimetry (DSC) measurements were performed on a differential scanning calorimeter (N-DSC II) supplied by Calorimetry Sciences Corp (Utah, USA). A pressure of 2 atm was applied to prevent the formation of air bubbles and avoid changes in the sample volume. The thermograms of 1:5.5 molar ratio of α-Lac:DOX and 1:0.5 and 1:5.5 molar ratios of α-Lac:PTX systems were recorded over a temperature range of 15-60 °C with a heating rate of 2 °C/min. The reference cell was loaded with Tris buffer. All experiments were performed in a solution of 1.5 mg/ml α-Lac in Tris buffer containing EDTA. The sample scans were prepared for analysis by subtracting the buffer baseline followed by calculation of thermodynamic parameters from thermograms using CpCalc software (version 2.1) supplied by the manufacturer.

### Circular dichroism spectroscopy

Far-UV (195-260 nm) and near-UV (260-320 nm) circular dichroism (CD) spectra were measured to evaluate changes in the secondary and tertiary structures, respectively, of α-Lac induced by DOX or PTX. The CD spectra were recorded at room temperature (25 °C) by an Aviv 215 Spectropolarimeter (Lakewood, New Jersey, USA) using a cuvette of 1 mm path length for far-UV and 2 mm path length for near-UV measurements. Apo α-lac was formed by dissolving α-Lac (300 µl, 10 µM) in Tris buffer (20 mM) containing EDTA (3.5 mM) to remove the bound Ca^2+^. Afterwards, α-Lac was titrated with small increments of DOX or PTX stock solutions (3.4 and 7 mM, respectively). At the end of titration, molar ratios of 1:2.0 and 1:5.5 were obtained for both α-Lac:DOX and α-Lac:PTX. The α-Lac:DOX and α-Lac:PTX solutions were vortexed for 15 s followed by incubation for 4 min to allow the formation of α-Lac:DOX and α-Lac:PTX complexes prior to CD measurements. Due to DOX and PTX absorption in the wavelength range of experiments, it was necessary to do a correction. Therefore, CD spectra were corrected by subtracting the spectra of DOX or PTX from α-Lac in the presence of various concentrations of DOX or PTX^56,57^. The CD results were expressed in terms of molar ellipticity ([θ]) in deg cm^2^ dmol^−1^ according to the following equation:

5 $$[{\rm{\theta }}]=100\times 115.3\times {\rm{\theta }}/{\rm{cl}}$$

where 115.3 is the mean amino acid residual weight (MRW) for bovine α-Lac^55^, θ is the CD signal in degrees at each wavelength, c is the α-Lac concentration in mg/ml and l is the length of the lightpath (cm). CDSD and CDNN software analysis was applied to deconvolute the CD spectra to determine the tertiary structure and the content of various secondary structure elements.

### Pace analysis to determine protein thermal stability

The stability of α-Lac upon interaction with DOX or PTX was analyzed according to the two-state model of Pace^46^. Briefly, thermal denaturation curves of 10 μM α-Lac solution in the presence and absence of 2.0-fold and 5.5-fold concentrations of DOX or PTX (3.4 and 7 mM, respectively) were obtained by Carry Eclipse (Varian, Australia) spectrofluorimeter, at excitation and emission wavelengths of 280 and 337 nm, respectively. α-Lac and the drugs mixed solutions in Tris buffer were vortexed for 15 s, followed by 4 min incubation. Afterwards, α-Lac solution in complex with DOX or PTX was scanned in the temperature range of 20 to 95 °C. Temperature was raised 2 °C per min and the data were recorded every 1 min. The fluorescence intensity was corrected according to the equation (6) to remove the absorption of different concentrations of DOX or PTX in the same temperature range^58^:

6 $${\rm{F}}={{\rm{F}}}_{{\rm{Init}}{\rm{.}}}\times {10}^{((Aex+Aem)/2)}$$

where F and F_Init_ represent corrected and initial fluorescence intensity, respectively. A_ex_ represents absorption of DOX or PTX at the excitation wavelength and A_em_ is absorption of DOX or PTX at the emission wavelength.

### Lifetime measurements

Fluorescence lifetime measurements were performed using an Edinburgh Instruments (FLS 920) spectrofluorimeter in laser mode at excitation and emission wavelengths of 255 and 337 nm, respectively. The spectrofluorimeter was equipped with a temperature-controlled cell connected to a circulating water bath. Fluorescence lifetime of 10 µM α-Lac in 20 mM Tris buffer (pH 7 and containing EDTA) in the presence and absence of 2.0- and 5.5-fold concentrations of DOX or PTX were measured. Using a double-exponential decay function of the decay profiles with F900 analysis software, the lifetime values were determined from the reconvolution fit analysis. When applying the reduced χ^2^ value, the goodness of fit was assessed (close to 1 in all cases).

Furthermore, according to the following equation and using the amplitude α_i_ of the ith component lifetime τ_Fi_, the average fluorescence lifetime (τ_F_) was calculated since TRP and α-Lac presented two different lifetime values^59^:

7 $${\tau }_{{\rm{F}}}=\frac{{\sum }_{i}{\alpha }_{i}{\tau }_{Fi}^{2}}{{\sum }_{i}{\alpha }_{i}{\tau }_{Fi}}$$

### Fluorescence anisotropy measurements (A)

α-Lac labeling with FITC was performed according to published procedures^48^. In brief, α-Lac was dissolved in Tris buffer (pH 7.0) and reacted with FITC in a darkened lab. The FITC was first dissolved in a few drops of DMSO, then made up to volume in pH 7.0 Tris buffer. The sample was left on a shaker to react for 8 h at room temperature in the dark. Free unreacted dye was separated from the reaction mixture by repeated dialysis against 0.01 M buffer. Dialysis membranes (cut-off, 12000 Da) were prepared according to standard methods and immediately used. Dialysis was considered complete when free FITC fluorescence in the outer solution was no longer detectable.

Fluorescence spectra and steady-state emission anisotropies of all α-Lac samples were measured with Cary Eclipse fluorimeter (Agilent, Palo Alto, CA). Anisotropy was measured with a manual polarizer and the G factor correction was done manually, with Ludox® as the reference fluorophore. The labelled protein was then excited at 490 nm and the parallel and perpendicular emissions were monitored at maximum emission of 512 nm with a manual polarizer. The anisotropy measurement of the protein was then repeated with DOX or PTX. DOX is fluorescent and it absorbs light at 490 nm but has no fluorescence emission at 512 nm and shows no interference with the anisotropy measurements.

### Molecular docking

Molecular docking was performed to study the interactions between α-Lac and DOX or PTX using AutoDock Vina 1.1.2 program^60^. Blind docking was used to predict preferred orientation of ligands and binding affinities. Crystal structure of α-Lac was obtained from RCSB protein data bank (1HFZ PDB code). Afterwards, hydrogen atoms were added to the protein structure and initial files were prepared by MGL tools (1.5.6rc3 version)^61^. Furthermore, DOX and PTX structures were obtained from PubChem (https://pubchem.ncbi.nlm.nih.gov/) and ChemSpider (http://www.chemspider.com/), respectively. Besides, Kollman and Gasteiger charges were assigned to α-Lac and ligand molecules, respectively. Moreover, a grid volume that was big enough to cover the entire surface of the protein was applied using a grid spacing with value of 1 Å for blind docking. Docking results were clustered and the best mode was selected to use for local docking in which a Grid box of 30×30×30 points was used with a spacing of 1 Å. Exhaustiveness was set on 20 and the root mean square (RMS) cluster tolerance was set to 2.0 Å. For other docking parameters, default values were applied. α-Lac was set to be rigid while DOX or PTX molecules were set to be flexible through both blind and local docking. The graphical images representing the best pose were prepared using MGL tools. Besides, LigPlot+ v.1.4.5, was employed to present schematic two dimensional representations of protein-ligand interactions using the PDB files as input^62^.

### Molecular dynamics simulation

The best docking poses of α-Lac:DOX and α-Lac:PTX complexes were considered as the initial structures for MD simulations. Restrained electrostatic potential (RESP) charges were assigned to the DOX and PTX molecules using the antechamber module from the AmberTools 14 suite^63^. The MD simulations were carried out with the GROMACS package (Version 5.1.3)^64^. Furthermore, the force field parameters for DOX and PTX were generated using antechamber module with the general AMBER force field (gaff), while the ff14SB force field was used for α-Lac^65,66^. The starting structures of α-Lac:DOX and α-Lac:PTX complexes were put in a periodic rectangular box with a closeness parameter of 12 Å. Then, TIP3P water molecules filled the simulation boxes^67^. Sodium counter ions were added to the systems to preserve the electroneutrality condition. Moreover, the particle mesh Ewald (PME) method was used to treat the long-range electrostatic interactions^68^. Besides, the SHAKE algorithm was used to constrain all bonds involving hydrogen atoms^69^. In addition, the initial velocities were taken from a Maxwell–Boltzmann distribution at a temperature of 300 K. Moreover, a cutoff distance of 10 Å was used to truncate van der Waals interactions in each simulation. The equations of motion were integrated using the leap frog algorithm^70^ with a time step of 2 fs. For each system, at first, the energy minimization was performed in 5000 steps, using the steepest descent method to remove bad contacts. During the minimization, position restraints with force constants of 1,000 kJ mol^−1^ nm^−2^ were considered on all heavy atoms of the α-Lac, DOX, and PTX molecules. Following the energy minimization, heating step was performed from 0 to 300 K through an NVT ensemble MD simulation for 1 ns. Afterwards, 1 ns equilibration was carried out at a constant temperature (300 K) and pressure (1 bar). The control parameters such as total energy, density, temperature, and RMSD were checked during the equilibration step, showing that the equilibration time is appropriate for performance of the next step. Finally, a 100-ns production run was carried out in the NPT ensemble. In NPT MD simulations, the temperature and pressure were controlled by the Nose´–Hoover thermostat^71^ and Parrinello–Rahman barostat^72^, respectively. Moreover, the coupling time of 0.1 and 1.0 ps was used for thermostat and barostat, respectively. Totally, three MD simulations were done (α-Lac:DOX and α-Lac:PTX complexes and free α-Lac). The atomic coordinates were saved every 25 ps for analysis. Moreover, molecular graphics were made with the PyMOL program (The PyMOL Molecular Graphics System, Version 1.7.2).

### Cell proliferation assay

The cytotoxicity of α-Lac, DOX, PTX, α-Lac:DOX, and α-Lac:PTX was determined using MTT assay. In this assay, harvested exponentially growing MDA-MB-231 and T47D cells were plated in 96-well plates. Afterwards, the cells were treated with freshly prepared solutions containing various concentrations of α-Lac alone (1-1000 μM), DOX alone (1, 2, 4, 10, and 20 μM), PTX alone (1, 2, 4, 10, and 20 μM), and α-Lac (1, 2, 4, 10, and 20 μM) along with DOX or PTX (1, 2, 4, 10, and 20 μM) with equal (1:1, 2:2, 4:4, 10:10, and 20:20) and ascending ratios (1:2, 1:4, 1:10, and 1:20). The corresponding control cells were also considered in the absence of any kind of treatments. The solutions were vortexed before treatment in order to trigger the interaction between α-Lac and DOX or PTX. After incubation for 24, 48, and 72 h, 10 μl of MTT (5 mg/ml) dissolved in PBS was added to each well followed by incubation for 3 h. Then, 100 μl DMSO was added to each well to dissolve the formazan crystals formed by viable cells. Finally, the absorbance of each well was measured at a test wavelength of 570 and a reference wavelength of 630 nm using an Elisa reader spectrophotometer (BioTek, USA). Cell viability was calculated by comparing absorbance of the treated cells with that of the control cells, which conventionally was 100%. The results were presented as means of three independent experiments^52^.

### Apoptosis assay

Apoptosis induction in absence and presence of α-Lac alone (1 and 2 μM), DOX alone (1 and 2 μM), PTX alone (1 and 2 μM), and α-Lac (1 and 2 μM) along with DOX or PTX (1 and 2 μM) with equal (1:1 and 2:2) and ascending (1:2) ratios was detected and quantified by flow cytometric analysis of treated MDA-MB-231 and T47D cells stained with acridine orange/ethidium bromide (AO/EB) according to the published procedures^73,74^. Following incubation for 24 or 48 h, the treated cells were trypsinized and pelleted. Afterwards, they were resuspended in 1 ml PBS and stained with AO and EB with the final concentration of 0.1 and 0.25 mM, respectively. All samples were analyzed by CyFlow Space (Partec, Germany) followed by data analysis using FloMax software.

### Statistical analysis

Statistical analyses were performed using SPSS version 16.0 statistical software (SPSS Inc., Chicago, USA). One-way analysis of variance (ANOVA) and Tukey’s post-hoc testing were applied to compare means for continuous variables. A two-tailed *p* value of <0.05 was considered statistically significant.

## Electronic supplementary material

Supplementary information
