# Supplementary material for: A biophysical study on the mechanism of interactions of DOX or PTX with α-lactalbumin as a delivery carrier
Source: Sci Rep. 2018 Nov 26;8:17345. doi: 10.1038/s41598-018-35559-1 (PMC6255783; doi:10.1038/s41598-018-35559-1)
Supplement: Supplementary file 1 — Supplementary information [file 41598_2018_35559_MOESM1_ESM.pdf]

A biophysical study on the mechanism of interactions of DOX or PTX with α-lactalbumin as a delivery carrier

Behdad Delavari, Fatemeh Mamashli, Bahareh Bigdeli, Atefeh Poursoleiman, Leila Karami, Zahra Zolmajd-Haghighi, Atiyeh Ghasemi, Samaneh Samaei-Daryan, Morteza Hosseini, Thomas Haertlé, Vladimir I. Muronetz, Øyvind Halskau, Ali Akbar Moosavi-Movahedi, Bahram Goliaei, Ali Hossein Rezayan, Ali Akbar Saboury

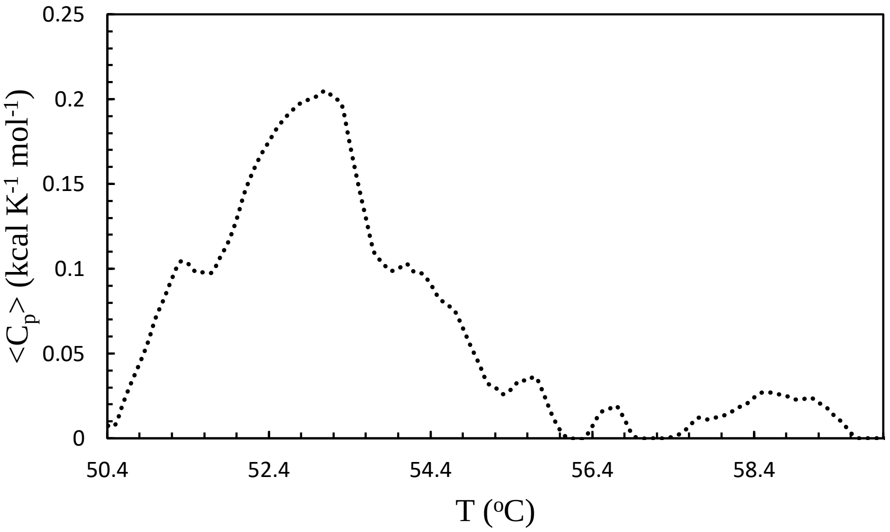

Figure 1. DSC thermograms of apo α-Lac in the presence of 0.86 mM PTX. DSC thermograms of 3 mg/ml apo α-Lac in 20 mM Tris buffer (pH 7) containing 3.5 mM EDTA in the presence of 0.86 mM PTX. The measurements were performed over a temperature range of 15-60 °C.

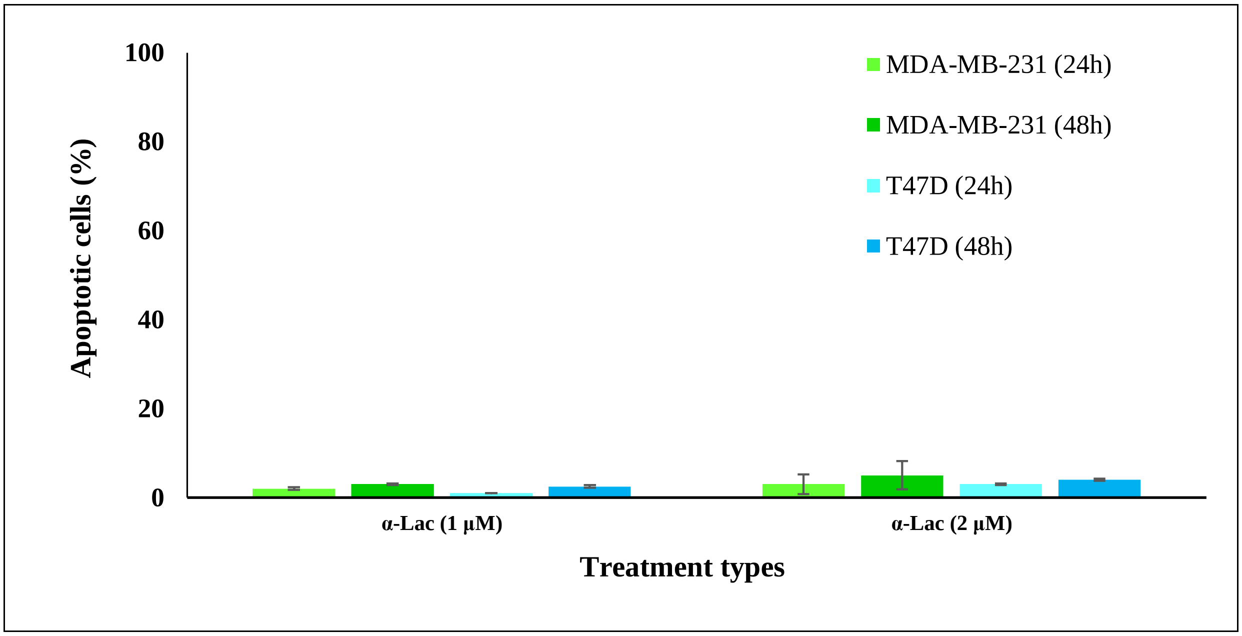

Figure 2. Effect of α-Lac on apoptosis induction in MDA-MB-231 and T47D cells. The cells were incubated with 1 and 2 µM α-Lac for 24 and 48 h. Apoptosis induction was evaluated by flow cytometry. Each data point is an average ± SEM of three independent experiments.

Table 1. Secondary structure changes of α-Lac upon interactions with various concentrations of DOX or PTX.

Sample	α-Helix	Antiparallel	Parallel	β-turn	Random Coil
α-Lac	34.2	9.5	8.4	18.1	29.8
α-Lac:DOX (1:2)	30.2	9.3	9.3	18	32.5
α-Lac:DOX (1:5.5)	28.5	9.6	9.6	17.9	33.8
α-Lac:PTX (1:2)	34.4	8.5	8.5	17.8	30.2
α-Lac:PTX (1:5.5)	29.5	9.4	9.4	17.8	33.1
